# Hydrodynamic and kinetic study of a hybrid detoxification process with zero liquid discharge system in an industrial wastewater treatment

**DOI:** 10.1186/s40201-014-0145-z

**Published:** 2014-12-24

**Authors:** Mohammad Fadhil Abid, Amir Aziz Abdulrahman, Noor Hussein Hamza

**Affiliations:** Chemical Engineering Department, University of Technology, Baghdad, Iraq

**Keywords:** Solar photocatalysis, Membrane separation, Hybrid system, Zero liquid discharge, Titanium dioxide photocatalyst, Dye synthetic wastewater, Falling-film slurry reactor

## Abstract

This work focused on the degradation of toxic organic compounds such as methyl violet dye (MV) in water, using a combined photocatalysis/low pressure reverse osmosis (LPRO) system. The performance of the hybrid system was investigated in terms of the degradation efficiency of MV, COD and membrane separation of TiO_2_. The aim of the present study was to design a novel solar reactor and analyze its performance for removal of MV from water with titanium dioxide as the photocatalyst. Various operating parameters were studied to investigate the behavior of the designed reactor like initial dye concentration (C = 10-50 mg/L), loading of catalyst (C_TiO2_ = 200-800 mg/L), suspension flow rate (Q_L_ = 0.3-1.5 L/min), pH of suspension (5–10), and H_2_O_2_ concentration (C_H2O2_ = 200-1000 mg/L). The operating parameters were optimized to give higher efficiency to the reactor performance. Optimum parameters of the photocatalysis process were loading of catalyst (400 mg/L), suspension flow rate (0.5 L/min), H_2_O_2_ concentration (400 mg/L), and pH = 5. The designed reactor when operating at optimum conditions offered a degradation of MV up to 0.9527 within one hours of operation time, while a conversion of 0.9995 was obtained in three hours. The effluent from the photocatalytic reactor was fed to a LPRO separation system which produced permeate of turbidity value of 0.09 NTU which is closed to that of drinking water (i.e., 0.08 NTU). The product water was analyzed using UV-spectrophotometer and FTIR. The analysis results confirmed that the water from the Hybrid-System could be safely recycled and reuse. It was found that the kinetics of dye degradation was first order with respect to dye concentration and could be well described by Langmuir-Hinshelwood model. A power-law based empirical correlation was developed for the photocatalysis system, related the dye degradation (R) with studied operating conditions.

## Introduction

Pollution of ground water and rivers by organic pollutants is a constant concern and a common problem throughout the world. Some of these organic pollutants are; herbicides, pesticides, and fungicides used in agricultural activities, which can be carried away by rain and polluted water resources, hydrocarbons from wastewater discharges from oil production activities and synthetic dyes from textile industry’s waste processing fluids [1]. Textile dyes and other commercial colorants have become as toxic organic compounds the focus of environmental remediation efforts because of their natural biodegradability is made increasingly difficult owing to the improved properties of dyestuffs [2]. Color interferes with penetration of sunlight into the water, retards photosynthesis, inhibits the growth of aquatic biota and interferes with solubility in water bodies [3]. Various physical, chemical and biological pre-treatment and post-treatment techniques have been developed over the last two decades to remove color from dye contaminated wastewater in order to cost effectively meet environmental regulatory requirements. Chemical and biological treatments have been conventionally followed till now but these treatment methods have their own disadvantages. The aerobic treatment process is associated with production and disposal of large amounts of biological sludge, while wastewater treated by, anaerobic treatment method does not bring down the pollution parameters the satisfactory level [4]. As international environmental standards are becoming more stringent, (ISO 14001, 1996), a technological system for the removal of organic pollutants, such as dyes has to be developed. Heterogeneous photocatalysis is one of the advanced oxidation process (AOP) that has proven to be a promising method for the elimination of toxic and bio-resistant organic and inorganic compounds in wastewater by transforming them into innocuous species [5,6]. Advanced oxidation process (AOP) is a chemical oxidative process, which can be applied to wastewater treatment to oxidize pollutants. It generates hydroxyl radicals which considered as the second strongest known oxidant (2.8 V vs. standard hydrogen electrode). It is able to oxidize and mineralize almost every organic molecule, yielding CO_2_ and inorganic ions [7]. AOPs not only oxidized the organic compounds, but also a complete mineralization is achievable, and the processes are not specific and therefore are capable of destroying a broad range of organic compounds. The process is very powerful, and is immune to organic toxicity. In the eighties and nineties, water reuse started to become a popular means to reduce freshwater intake and reduce treatment costs. A concept that refers to closed circuits of water, such that disposal is eliminated. Advantages and disadvantages of zero discharge facilities are currently being seriously considered and discussed. Zero liquid discharge minimizes the consumption of freshwater to that of make-up; therefore, it should help relieve freshwater availability limitations in places where it is scarce or expensive [8]. In photocatalytic degradation of dyes in wastewaters, the main operating parameters which affect the process are pH of the solution to be degraded, oxidizing agent, catalyst loading, and contaminant concentration [9]. Photocatalytic reactions are the result of the interaction of photons having the appropriate wavelength with a solid semiconductor [10]. Recent studies have been devoted to the use of photocatalysis in the removal of dyes from wastewaters, particularly, because of the ability of this method to completely mineralize the target pollutants [11].

The general mechanism of photocatalysis could be represented elsewhere [12-14]. Many semiconductors have been tested so far as photocatalysts, although only TiO_2_ in the anatase form seems to have the most interesting required attributes; such as high stability, good performance and low cost [15]. In this respect, the photodecomposition power of TiO_2_, for a wide variety of organic compounds present in water, has been reported in the literature [16].

Photocatalytic reactors for water treatment can generally be classified into two main configurations, depending on the deployed state of the photocatalysts: (1) reactors with suspended photocatalyst particles and (2) reactors with photocatalyst immobilised onto continuous inert carrier [17]. Various types of reactors have been used in the photocatalytic water treatment, including the annular slurry photoreactor [18], cascade photoreactor [19], downflow contactor reactor [20] and, etc. The disparity between these two main configurations is that the first one requires an additional downstream separation unit for the recovery of photocatalyst particles while the latter permits a continuous operation. Vincenzo et al. [21] carried out a photodegradation of two common and very stable azo-dyes, in aqueous suspensions of polycrystalline TiO2 irradiated by sunlight using a plug flow reactor in a total recirculation loop. They reported that Complete decolourization was obtained in few hours for both dyes but mineralisation occurred after longer times with the formation of CO2, nitrates and sulphates. Damszel et al. [22] investigated the possibility of application of the hybrid photocatalysis/membrane processes system for removal of azo dyes (Acid Red 18, Direct Green 99 and Acid Yellow 36) from water. The photocatalytic reactions were conducted in the flow reactor with immobilized photocatalyst bed and in the suspended system integrated with ultrafiltration (UF). They found that the solutions containing the model azo dyes could be successfully decolorized during the photocatalytic processes applied in the studies. The application of UF process results in separation of photocatalyst from the treated solutions whereas during the (NF) and membrane distillation (MD) high retention of degradation products was obtained.

The main objectives of this work were the evaluation and testing of a novel photocatalytic reactor for the degradation of methyl violet (MV) dye which is selected as a model organic toxic pollutant in water using a commercial TiO_2_ catalyst. And also to study the possibility to couple a membrane separation system with the reaction system, to remove TiO_2_ particles from product stream of the solar photocatalytic reactor to achieve a zero liquid discharge.

## Materials and methods

### Chemicals

Methyl violet 6B (MV) dye (commercial grade, C_24_H_28_N_3_Cl, *λmax* (nm) = 586, Sigma Aldrich Co., USA.), Titanium dioxide powder (antase type, ≥99.5% trace metals basis, particle size ~ 21 nm, specific surface area (35–65 m^2^/g), particle density (4.26 g/mL (at 25°C)), Sigma Aldrich Co.) were used as received. Reagent-grade hydrogen peroxide (H_2_O_2_) (50% v/v solution), was used as oxidant. Technical grade hydrochloric acid (35%) and sodium hydroxide (98% flakes) were used to adjust the pH of synthetic wastewater (to around 5–10). Distilled water (conductivity <10 μS cm^−1^, Cl^−^ = 0.7–0.8 mg L^−1^, NO3^−^ = 0.5 mg L^−1^, organic carbon <0.5 mg L^−1^).

### Analytical determinations

**Figure 1:**
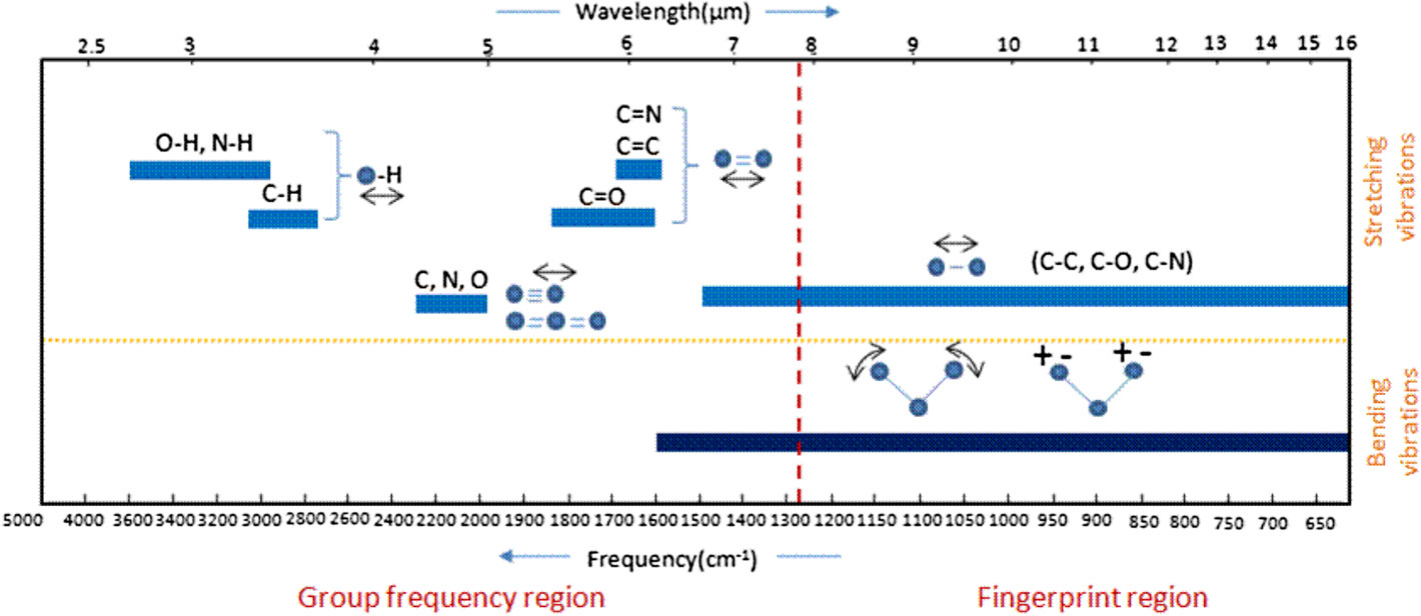
**Group frequency and fingerprint regions of the mid-infrared spectrum** **[** **http://chemwiki.ucdavis.edu/Physical-Chemistry/Spectroscopy** **].**

**Figure 2:**
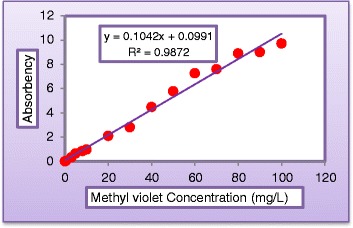
**Calibration curve of methyl violet dye.**

**Figure 3:**
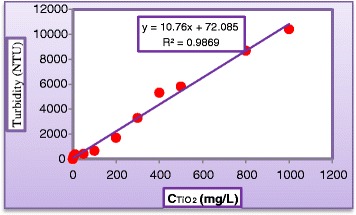
**Calibration curve of Turbidity for various TiO**_**2**_ **loadings in water.**

Mineralization was followed by measuring, the color which is a function of concentration was determined spectrophotometrically at a dominate wave length by method no. 2120 Standard Method, using a Shimadzu UV -Visible spectrophotometer (UB_1201 PC). COD was determined by open reflux method 5220 Standard Method (ET 108). FTIR (Bruker Tensor 27) system was used to identify the functional groups in product solutions with aid of (Figure 1). Turbidity of water treated by membrane system (LPRO) was measured by Turbid Direct meter (Lovibond). The sunlight intensity was measured by using Davis 6152C Vantage Pro2 Weather Station radiometer. Table 1 shows a sample of incident solar radiation measurements. Calibration curves of dye concentration vs. light absorbency and TiO_2_ concentration vs. turbidity were illustrated in (Figures 2 and 3), respectively.

**Table 1:** **Average daily incident solar radiation (kWh m** ^**−2**^ **) on 37° inclined surface at University of Technology-Baghdad**

**No.**	**Average daily global incident solar radiation, kWh. m** ^**−**^ **2**	**Average daily UV incident solar radiation, kWh.m** ^**−2**^	**Month**
1	5.7	0.456	May
2	6.8	0.544	June
3	5.7	0.456	July
4	4.5	0.36	August
5	5.9	0.472	September

### Experiment set-up

**Figure 4:**
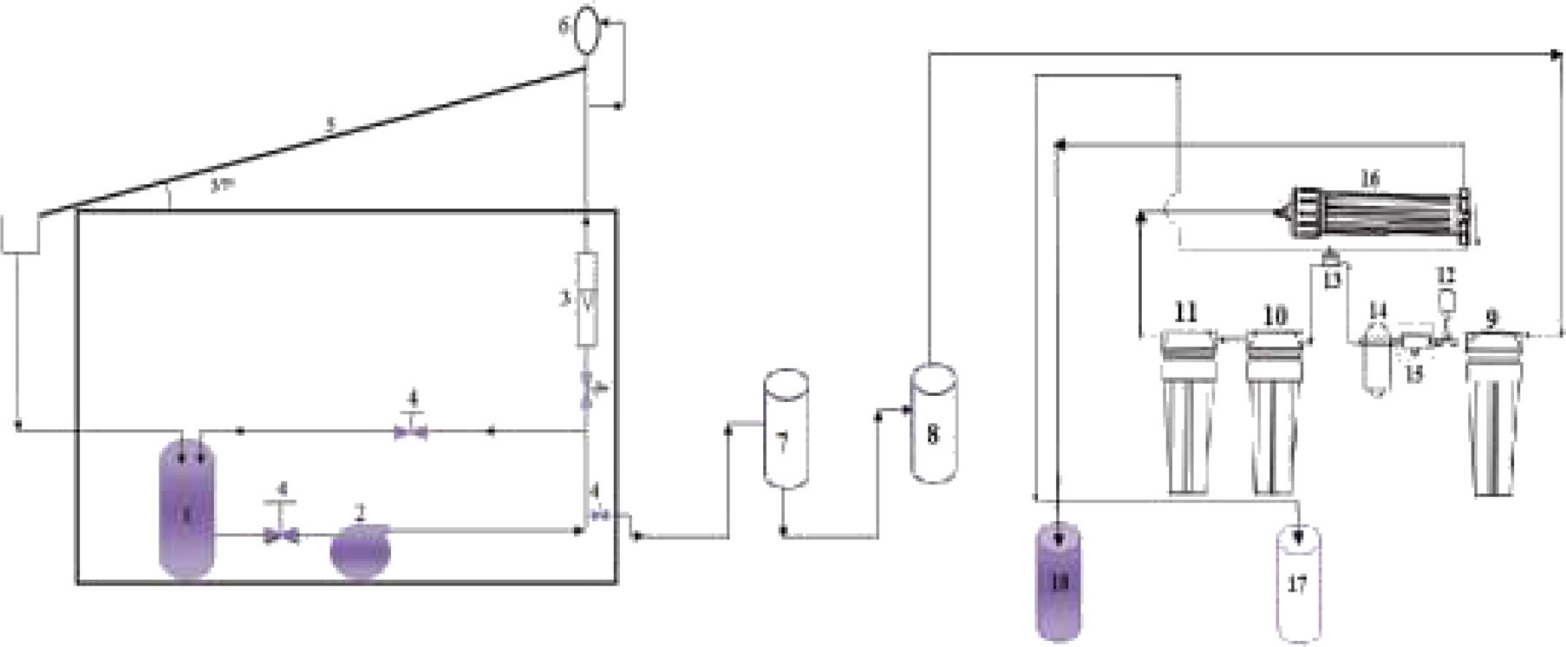
**1- Feed tank to reactor; 2- Feeding pump; 3- Rotameter; 4- Valve; 5- Flate plate reactor; 6- Liquid distributo; r 7- Neutralization tank; 8- Feed tank to membrane; 9- 5micron PP filter; 10&11- Buffer vessels; 12- Low pressure switch 13- Auto shut off 14- Booster pump; 15- Inlet solenoid valve; 16- LPRO membrane; 17- Permeate tank; 18- Concentrate tank.**

The hybrid process setup consists of two treatment systems connected together. Figure 4 shows the schematic view of the (photocatalysis reaction/LPRO membrane) system. The photocatalytic reactor was operated as a batch process. The system consists of a solar reactor (no.5), wastewater preparation tank (no.1) made of 5 L-PVC, a circulation pump (no.2), Type: ln–line centrifugal pump (Wtg204), Head (H) = (25–40) m, variable impeller speed (750, 1200, 1850 min^−1^), V = 220 volt). The solar reactor was mounted on a fixed platform tilted 37° (local latitude) and directed south-east. It was made up of a flat-plate colorless glass of dimensions 1000 × 750 × 4 mm. The base of the reactor was made of aluminum. This geometry enables the light entering the liquid film from almost any direction to be reflected and can also be employed for the photocatalytic reaction. The circulating pump was used to feed the water from the tank to the reactor via a calibrated flow meter (no.3). The aqueous solution was allowed to trickle down freely from a pipe (no.6) pierced by several openings placed at the top of the reactor. The water and reagents added to the tank from openings in the lid. A thermocouple type (pt-100) was placed into the water preparation tank to measure the mixture temperature. A mechanical mixer was used to obtain homogeneous conditions in the water tank. In the present work, the pH of the effluent from the photocatalytic reactor was neutralized in a 5 L vessel (no.7) and then left for 4 hours. The sediment was washed with H_2_O_2_, dried, and weighted for further use. The neutralized solution was fed to a 5 L PVC tank (no.8) which served as a feeding tank to the low pressure reverse osmosis membrane (LPRO) type (RE1812-CSM Co.) which was used to separate the TiO_2_ nanoparticles from the photoreactor effluent via a diaphram pump (no.14), (type CR50-N-N-2, single phase: 50 Hz, 220 V). The (LPRO) was a spiral wound module made of composite polyamide with an effective area of 0.7 m^2^. The separation system also contained two holding tanks, all are made of PVC, these are the concentrate tank (no.18), and the permeate tank (no.17). Each tank is supplied with suitable fittings and connections to serve the process. Laboratory portable conductivity and pH meters from Hanna-USA were used for further check and quick measurements.

### Experimental design

Photocatalytic reactor experiments were aimed to study the effect of operating parameters (catalyst loading, hydrogen peroxide concentration, flow rate, pH, and dye concentration) on the degradation efficiency and COD removal of organic pollutant. Table 2 shows the range of operating variables that used in photocatalytic experiment.

**Table 2:** **Range of operating variables in photocatalytic experiment**

**Operating parameter**	**Range**
Catalyst loading (TiO_2_), mg/L	200, 300, 400, 500, 800
Hydrogen peroxide concentration, mg/L	300, 400, 500, 800, 1000
Flow rate, L/min	0.3, 0.5, 1, 1.5
pH	5, 6, 7, 9, 10
Dye concentration, mg/L	10,20, 30, 40, 50

## Results and discussion

### Effect of operating parameters on dye degradation and COD removal in photocatalytic reactor

#### Effect of pH

**Figure 5:**
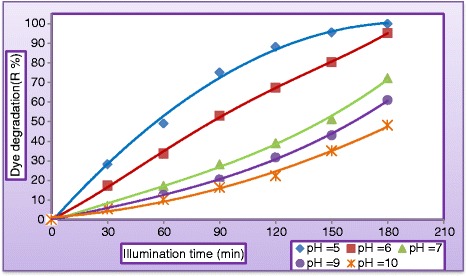
**Variation of dye degradation against illuminated time at different pH (C**_**MV**_ **= 30** **mg/L** **, C**_**TiO2**_ **= 400 mg/L, C**_**H2O2**_ **= 400 mg/L, and Q**_**L**_ **= 0.5 L/min).**

**Figure 6:**
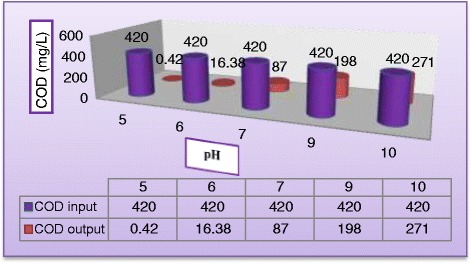
**Effect pH on COD removal (C**_**MV**_ **= 30** **mg/L** **, C**_**TiO2**_ **= 400 mg/L, C**_**H2O2**_ **= 400 mg/L, and Q**_**L**_ **= 0.5 L/min) after 180 min.**

pH is an important parameter for the photocatalytic process, and it is of interest to study its influence on the degradation rate of the MV dye. (Figures 5 and 6) illustrate the variation of dye degradation rate against illuminated time and the variation of COD in reactor effluent against pH after 180 min, respectively. Results obtained experimentally by varying initial pH of polluted solution from 5 to 10 with keeping all other parameters unchanged at (C_MV_ = 30 mg/L, C_TiO2=_ 400 mg/L, Q_L_ = 0.5 L/min) clearly indicated a neat decrease in dye degradation. It could be noticed from (Figure 5) that the final degradation obtained in acidic solution at pH equal 5 was 99.95% and at pH = 6 it was 95.22% while at pH = 7, pH = 9, and pH = 10, the final degradation efficiency were 72.2%, 60.98%, and 48.2%, respectively. At the same condition, Figure 6 indicated that COD removal was 99.9% after 180 min of operation. This could be explained from the surface charge of TiO_2_ point of view. In acidic pH, the surface of TiO_2_ acquires a positive charge thereby attracting the anionic MV dye, leading to a greater adsorption and hence increasing the degradation rate and COD removal in the acidic media. However, the reverse image is observed in the basic medium where the TiO_2_ surface was negatively charged which repels the dye molecules away from the surface of the catalyst thereby decreasing the degradation rate. The adsorption is maxima at pH 5 and so is the degradation rate and COD removal. The change of the surface properties of TiO_2_ with the change of pH values around its point of zero charge (pH_pzc_) is according to the following reaction [23]:

1 $$ \begin{array}{cc}\hfill \mathrm{TiOH}+{\mathrm{H}}^{+}\rightleftarrows {\mathrm{TiOH}}_2^{+}\hfill & \hfill \mathrm{p}\mathrm{H}<{\mathrm{pH}}_{\mathrm{pzs}}\hfill \end{array} $$

2 $$ \begin{array}{cc}\hfill \mathrm{TiOH}+{\mathrm{OH}}^{-}\rightleftarrows {\mathrm{TiO}}^{-}\hfill & \hfill \mathrm{p}\mathrm{H}>{\mathrm{pH}}_{\mathrm{pzs}}\hfill \end{array} $$

pH changes can thus influence the adsorption of dye molecules onto the TiO_2_ surface, an important step for the photooxidation to take place. For Degussa P25, the pH_pzc_ is around 5.8 to 6.9. So, when pH <6 a strong adsorption of MV on the TiO_2_ particles is observed as a result of the electrostatic attraction of the positively charged TiO_2_ with the dye. In our case the highest degradation efficiency was achieved at pH = 5. In alkaline solution, the MV molecules are negatively charged and their adsorption is also expected to be affected by an increase in the density of TiO^−^ groups on the semiconductor surface. Thus, due to Coulombic repulsion, substrate is scarcely adsorbed. At high pH values, hydroxyl radicals are so rapidly scavenged that they do not have the opportunity to react with dyes [24,25]. For the above reasons the photocatalytic activity of anionic dyes reached a maximum in acidic conditions followed by a decrease in the pH range 7–10 [26].

#### Effect of hydrogen peroxide (H_2_O_2_)

**Figure 7:**
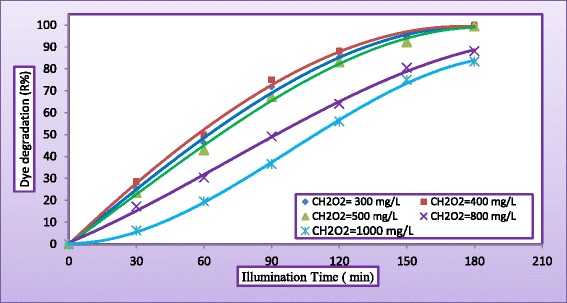
**Variation of dye degradation with illuminated time at different concentration of H**_**2**_ **O**_**2**_ **(C**_**MV**_ **= 30** **mg/L** **, C**_**TiO2**_ **= 400 mg/L, pH = 5, and Q**_**L**_ **= 0.5 L/min).**

**Figure 8:**
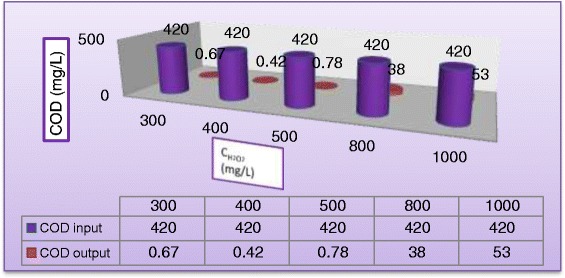
**Variation of COD removal in reactor effluent at different concentration of H**_**2**_ **O**_**2**_ **(C**_**MV**_ **= 30** **mg/L** **, C**_**TiO2**_ **= 400 mg/L, pH = 5, and Q**_**L**_ **= 0.5 L/min) after 180 min.**

**Figure 9:**
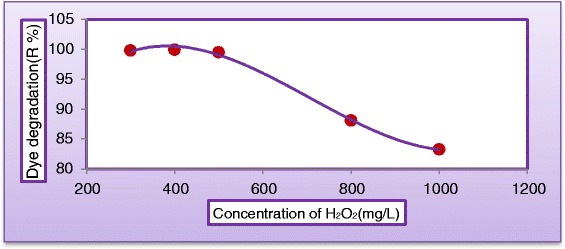
**Variation of dye degradation with concentration of H**_**2**_ **O**_**2**_ **(C**_**MV**_ **= 30** **mg/L** **, C**_**TiO2**_ **= 400 mg/L, pH = 5, and Q**_**L**_ **= 0.5 L/min) after 180 min.**

The addition of H_2_O_2_ may play an important role in the limitation rate of photogenerated hole–electron pairs which had been attributed to degradation rate and COD removal of the MV dyes. (Figures 7 and 8) plot the variation of dye degradation against H_2_O_2_ concentration in solution keeping all other parameters unchanged and COD removal in reactor effluent after 180 min at the same operating conditions, respectively. Different concentrations of H_2_O_2_ (300 to 1000 mg/L) were added to study the effect of H_2_O_2_ concentration on the decolorization rate and COD removal. As can be seen, the removal rate increased with increasing initial concentration of H_2_O_2_. The decolorization rate was slow at low H_2_O_2_ concentration, as the formation of hydroxyl radicals was insufficient, this may be explained the ability of H_2_O_2_ to trapping the electrons preventing the electron–hole recombination and hence increasing the chance of formation of OH^•^ radicals on the surface of the catalyst [27]. However, as the initial concentration of H_2_O_2_ increased beyond a certain value (400 mg/L, the increasing in decomposition rate becomes less. This because at higher H_2_O_2_ concentration, more OH^•^ was produced leading to a faster oxidation rate. However, these excess free radicals preferred to react with the excess of H_2_O_2_ rather than with the dye [28]. As shown in Figure 8, the addition of H_2_O_2_ (400 mg/L) to the dispersed solution resulted in a significant increase on the removal of COD. At the end of 180 min. of irradiation, almost total removal (99.5%) was obtained. When H_2_O_2_ concentration was increased to 1000 mg/L, COD reduction was only 87.4%. It seems that Hydrogen peroxide played a dual role in photocatalytic reaction, it is acting as an electron acceptor and could decompose to produce OH^•^ radicals [6]. It is clear from Figure 9 that the rate of degradation goes on increasing with increased concentration of H_2_O_2_ and approached maxima at 400 mg/L and then started to decrease with further increase in concentration of H_2_O_2_. The optimum hydrogen peroxide concentration for the degradation of MV dye is 400 mg/L. This amount of H_2_O_2_ will be employed into all experiments. (Figure 5) through (Figure 9) depict that the decolourization rates under UV/H2O2 decreased with increasing pH. In alkaline condition, H2O2 will decompose into water and oxygen rather than hydroxyl radicals. This causes the lower decolourization rates of azo dyes at higher pH values because the concentration of OH- is reduced under these conditions. The decolourization rate was increasingly less effective at pH values higher than 8, while acidic conditions achieve a more effective decolourization These results were confirmed by the findings of [29-32].

#### Effect of liquid flow rate

**Figure 10:**
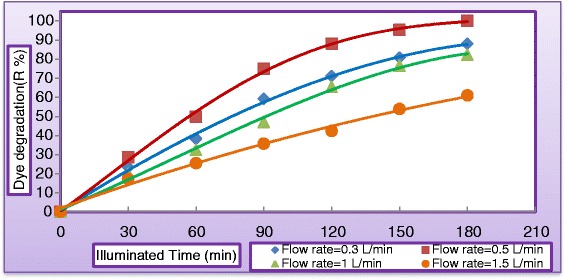
**Variation of dye degradation with illuminated time at different liquid flow rate (C**_**MV**_ **= 30** **mg/L** **, C**_**TiO2**_ **= 400 mg/L, pH = 5, and C**_**H2O2**_ **= 400 mg/L).**

**Figure 11:**
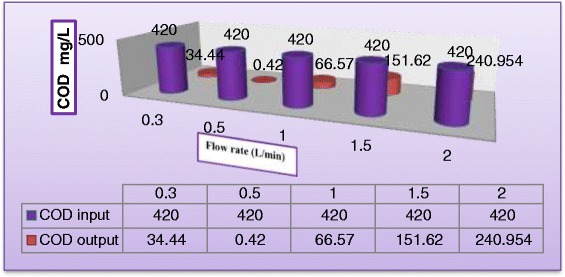
**Effect of liquid flow rate on COD removal in reactor effluent (C**_**MV**_ **= 30** **mg/L** **, C**_**TiO2**_ **= 400 mg/L, pH = 5, and C**_**H2O2**_ **= 400 mg/L) after 180 min.**

**Figure 12:**
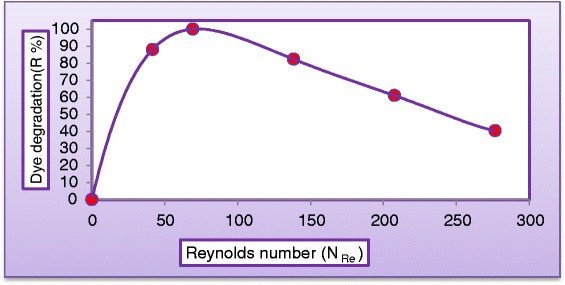
**Variation of dye degradation with liquid Reynolds numbers (C**_**MV**_ **= 30 mg/L, C**_**H2O2**_ **= 400 mg/L, C**_**TiO2**_ **= 400 mg/L, and pH = 5) after 180 min.**

Falling–film reactors characterized by a high ratio of exposure (surface area to liquid volume), which positively impact the performance of such type of reactors. Flow rate of synthetic wastewater is another important parameter which must be considered. Effect of liquid flow rate on the dye degradation and COD removal was tested by taking various flow rates from (0.3 to 1.5 L/min) keeping all other parameters unchanged at (C_MV_ = 30 mg/L, ${\mathrm{C}}_{{\mathrm{TiO}}_2}$ = 400 mg/L, ${\mathrm{C}}_{{\mathrm{H}}_2{\mathrm{O}}_2}$ = 400 mg/L, and pH = 5). Figure 10 illustrates the variation of the dye degradation against illuminated time. As can be seen from Figure 10 that liquid flow rate has negative impact on degradation rate. This may be explained from the view point of shorter contact time of aqueous suspension with illumination source as the recirculation rate increased. Figure 11 illustrates the influence of liquid flow rate on the COD of the reactor effluent. It can be concluded from the graph that effluent with 0.5 L/min has undergone almost complete degradation at 180 min of solar exposure which indicated that resulted water could be recycled in the process. When the liquid flow rate increased to 1.5 L/min, COD of the reactor effluent has dropped to 241 mg/L which indicates the necessity for further light exposure. This could be firstly due to the limitation of the solar light penetration because of rise in the liquid thickness and secondly to the reduction of the residence time of substrate which lead to reduce the surface reaction efficiency. Figure 12 shows dye degradation against Reynolds number of the liquid falling–film. The liquid Reynolds number *N*_*Re*_ can be calculated from equation (3) [33].

3 $$ {\mathrm{N}}_{\mathrm{Re}}=4{\mathrm{Q}}_{\mathrm{L}}{\uprho}_{\mathrm{L}}/{\mathrm{W}\upmu}_{\mathrm{L}} \cos \upbeta $$

where *Q*_*L*_ = Liquid flow rate (m^3^/s), *ρ*_*L*_ = Density of water (kg/m^3^), *W* = Width of reactor, *μ*_*L*_ = Dynamic viscosity of water (kg/m. s), and *β* = altitude angle. As can be seen, the impact of liquid Reynolds number on dye degradation shows a positively increasing trend to a point where all the surface of the photocataytic reactor was covered with a thin falling–film of synthetic wastewater, where dye degradation reported 99.95% at *N*_*Re*_ = 69.2 after then dye degradation started to decrease with further increasing of liquid flow rate at *N*_*Re*_ = 138.4, *N*_*Re*_ = 207.6, and *N*_*Re*_ = 276.6 the dye degradation were 82.3%, 64.4%, and 40.32%, respectively. This may be attributed to decreasing the residence time of reactants as the liquid flow rate increased.

#### Effect of initial dye concentration

**Figure 13:**
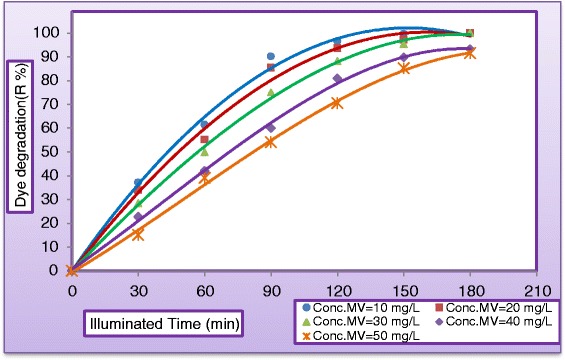
**Variation of dye degradation with illuminated time at different concentrations of MV dye (C**_**H2O2**_ **= 400 mg/L, C**_**TiO2**_ **= 400 mg/L, and pH = 5, Q**_**L**_ **= 0.5 L/min and pH = 5).**

The effect of initial concentration of dye solution on dye degradation efficiency has been investigated by varying the dye concentrations from (10 to 50 mg/L). Figure 13 plots the variation of dye degradation against (MV) dye concentration in the presence of 400 mg TiO_2_/L under solar light by keeping all other parameters unchanged. As can be seen from Figure 13 that after 180 min of irradiation time the degradation rate was 99.99%, 99.97%, 99.95%, 93.2%, and 91.52% at concentration of MV equal to (10, 20, 30, 40, and 50 mg/L), respectively. Dye degradation was observed to decrease as initial concentration increased. It could be concluded from the present experiments that as the dye concentration increases, the fraction of un-adsorbed dye in the solution increases, leading to lesser penetration of light through the solution onto the surface of TiO_2_, thereby decreasing the rate of formation of OH radicals, consequently the degradation efficiency decreased. However, the reverse image is observed at lower substrate concentration, where the light intensity and time of irradiation is same but interception of the photons to the catalyst surface is increased leading to the formation of more numbers of OH radicals, thereby increasing the rate of reaction. This concludes that as the initial concentration of the dye increases, the requirement of catalyst surface needed for the degradation also increases. Our results were confirmed by the findings of many researchers [25,26].

#### Effect of catalyst loading

**Figure 14:**
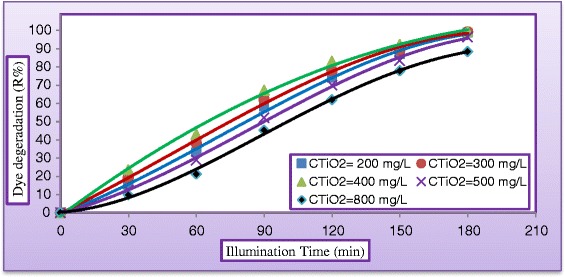
**Variation of dye degradation against time at different concentration of TiO**_**2**_ **(C**_**MV**_ **= 30 mg/L, C**_**H2O2**_ **= 500 mg/L, pH = 5, and Q**_**L**_ **= 0.5 L/min)**.

**Figure 15:**
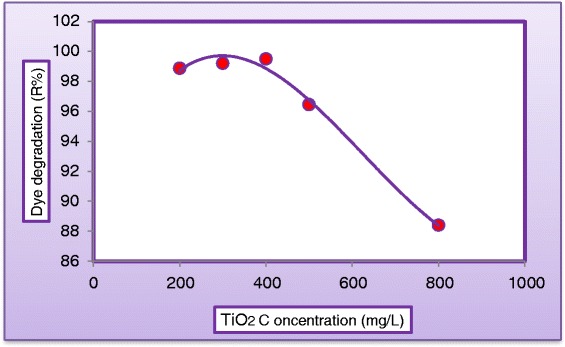
**Variation of dye degradation against concentration of TiO**_**2**_ **(C**_**MV**_ **= 30 mg/L, C**_**H2O2**_ **= 500 mg/L, pH = 5, and Q**_**L**_ **= 0.5 L/min) after 180 min.**

The amount of catalyst is one of the main parameters for the degradation studies from economical point of view. To investigate the effect of the photocatalyst, its loading in the dispersion was varied between (200 to 800 mg/L) under UV light. Figure 14 shows the variation of dye degradation (R %) against illuminated time for various photocatalyst loading by keeping all other parameters unchanged. As can be seen, at a specified catalyst loading the degradation efficiency appeared to increase rapidly at the first part of experimental period while the rate of increase seems to be slower at the second part. This may be due to the concentration of the dye which is higher at the beginning of the photodegradation process resulted in high rate of reaction and dye concentration decreases as the reaction proceeds. Figure 14 demonstrates a positive impact of catalyst concentration on dye degradation; this trend was almost due the increase of active site with absorption and dye adsorption. Our results were in agreements with the findings of [25,34]. In order to avoid the use of excess catalyst it is necessary to find out the optimum loading for efficient removal of dye. Figure 15 illustrates the optimum catalyst concentration for the degradation of MV dye after 180 min of treatment process. From Figure 15 it was observed that as catalyst concentration increased from (200 to 400 mg/L) the degradation rate increases correspondingly, this can be explained by the fact that there was an increased in the photon adsorption with increased concentration. The degradation rate decreased as the catalyst loading increased from (400 to 800) mg/L, this phenomenon may be explained by the light scattering, caused by the lightproof suspended catalyst [25,28].

## Photocatalysis kinetics

**Figure 16:**
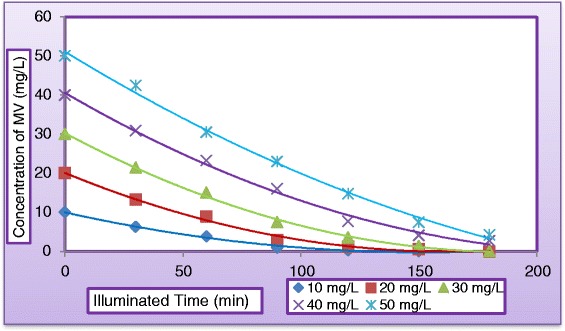
**Effect of initial concentration of MV on the Photocatalytic degradation at optimum operating conditions.**

The photocatalytic oxidation kinetics of many organic compounds has often been modeled with the Langmuir–Hinshelwood equation, which also covers the adsorption properties of the substrate on the photocatalyst surface. This model, developed by [35,36], is expressed as equation (4):

4 $$ \mathrm{r}=-\mathrm{d}\mathrm{C}/\mathrm{d}\mathrm{t}={\mathrm{k}}_{\mathrm{r}}\mathrm{K}\mathrm{C}/\left(1+\mathrm{K}\mathrm{C}\right) $$

where *r* is the reaction rate (mg/L.min), k_r_ the reaction rate constant (mg/L.min), *K* the adsorption equilibrium constant (L/mg) and C the concentration of dye (mg/L). The degradation rate of dye was studied as a function of the initial dye concentration in the range (10 to 50 mg/L), for a catalyst loading of TiO_2_ (0.4 mg/L). The results were illustrated in Figure 16 which shows the initial dye concentration versus reactor operating time. Equation (5) depicts a pseudo–first order reaction with respect to the methyl violet (MV) concentration. The relationship between the initial degradation rate (r_o_) and the initial concentration of organic substrate for a heterogeneous photocatalytic degradation process has been described by Langmuir–Hinshelwood model and can be written as follows: if we consider that the kinetic of dye degradation is of pseudo-order, at t = 0 and C = C_o_, equation (4) becomes:

5 $$ {\mathrm{r}}_{\mathrm{o}}={\mathrm{k}}_{\mathrm{r}}{\mathrm{KC}}_{\mathrm{o}}/\left(1+{\mathrm{KC}}_{\mathrm{o}}\right) $$

**Figure 17:**
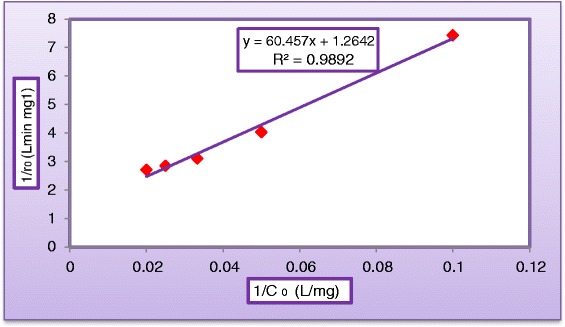
**Linearization of Langmuir–Hinshelwood’s equation of MV dye (C**_**TiO2**_ **= 400 mg/L, C**_**H2O2**_ **= 500 mg/L, pH = 5, and Q**_**L**_ **= 0.5 L/min).**

This Equation can be rearranged into linear form:

6 $$ 1/{\mathrm{r}}_{\mathrm{o}}=\left(1/{\mathrm{k}}_{\mathrm{r}}\mathrm{K}\right).1/{\mathrm{C}}_{\mathrm{o}}+1/{\mathrm{k}}_{\mathrm{r}} $$

where 1/r_o_ and 1/C_o_ are the dependent and independent variables, respectively, 1/k_r_ is the intercept and (1/k_r_K) is the slope of the straight line shown in Figure 17. The L-H adsorption constant and the rate constant were obtained using initial rate method [37] by plotting 1/r_o_ versus 1/C_o_. The values of the adsorption equilibrium constant, K and the kinetic rate constant of surface reaction, k_r_ were calculated. The graphical representation of equation (6) yields a straight line as shown in Figure 17 indicating a pseudo-first order reaction. The reaction rate constants k_r_ for photocatalytic degradation of dye were evaluated from experimental data Figure 17 using a linear regression. The constants k_r_ and K in Langmuir–Hinshelwood model were obtained as 0.791 mg/L. min and 0.0209 L/mg, respectively. The correlation coefficient R^2^ was equal to 0.9892 then equation (6) will become

7 $$ \mathrm{r}=0.0165\mathrm{C}/\left(1+0.0209\mathrm{C}\right) $$

## Empirical correlation

Dye degradation at each average seasonal temperature is the result of the interaction of several parameters, namely: catalyst concentration (C_TiO2_) Hydrogen peroxide concentration (C_H2O2_), pH of solution, liquid flow rate (Q_L_) and dye concentration (C_MV_). A power law formula was proposed to correlate the present experimental results with dye removal of Methyl violet dye. Equation (8) represents the proposed empirical correlation:

8 $$ \mathrm{R}={\mathrm{a}}_{\mathrm{o}}{\left({\mathrm{C}}_{\mathrm{TiO}2}\right)}^{\mathrm{a}1}{\left({\mathrm{C}}_{\mathrm{H}2\mathrm{O}2}\right)}^{\mathrm{a}2}{\left(\mathrm{p}\mathrm{H}\right)}^{\mathrm{a}3}{\left({\mathrm{Q}}_{\mathrm{L}}\right)}^{\mathrm{a}4}{\left({\mathrm{C}}_{\mathrm{MV}}\right)}^{\mathrm{a}5} $$

where: a_1_, a_2_, a_3_, a_4_, and a_5_ are constants, representing the magnitudes of the effect of applied catalyst concentration, hydrogen peroxide concentration, pH solution, liquid flow rate, and dye concentration, respectively on the objective function (i.e., dye degradation), while a_o_ is a constant which depends on the nature of the operating system. Regression analysis method using STAISTICA version 6.2 software was utilized to fit the experimental data for photocatalytic reactor with the proposed model gives the following equation with correlation factor of 0.9708 and variance equal to 0.95,

9 $$ \mathrm{R}=13.66{\left({\mathrm{C}}_{\mathrm{TiO}2}\right)}^{-0.07}{\left({\mathrm{C}}_{\mathrm{H}2\mathrm{O}2}\right)}^{-0.14}{\left(\mathrm{p}\mathrm{H}\right)}^{-0.92}{\left({\mathrm{Q}}_{\mathrm{L}}\right)}^{-0.47}{\left({\mathrm{C}}_{\mathrm{MV}}\right)}^{-0.04} $$

The coefficients of the correlation depict the predominant effect of pH on other operating variables for dye degradation. This correlation could be used as a model to predict the behavior of the dye degradation within the operating range of the studied variables.

### FTIR measurements

**Figure 18:**
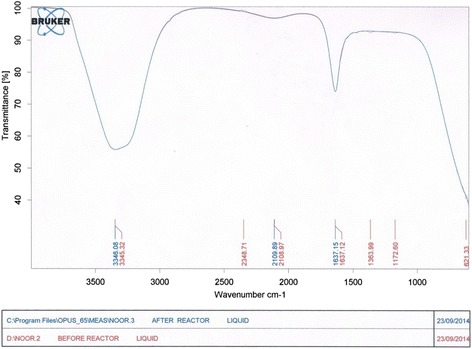
**Printout of FTIR analysis of samples at influent and effluent of solar reactor, respectively.**

**Figure 19:**
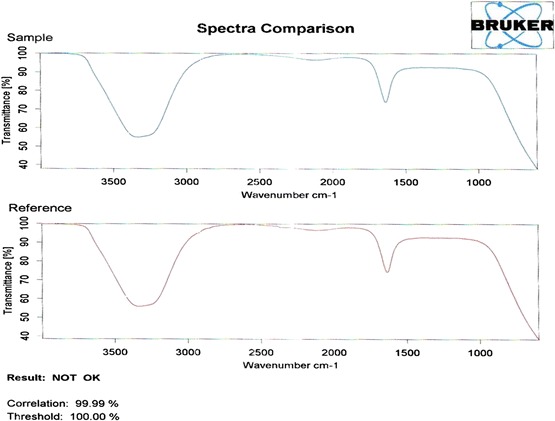
**Contrast of FTIR of MV dye compared with tap water after 180 min at optimum operating conditions.**

Samples were taken after 180 min from the reactor effluent analyzed with FTIR (type: Bruker Tensor 27) after neutralization and filtration with (0.25) μm flat paper. Figure 18 shows a printout of the FTIR instrument. The Figure illustrates two curves, the red one is for the contaminated sample with dye concentration of 30 mg/L. The blue curve is for the sample taken at the reactor effluent. Three small peaks corresponding to wavenumbers of 1172.6, 1363.99, and 2348.71 cm^−1^ appeared on the red curve represent functional groups of C-C, C-O, C-N, C, N, O. These groups were not found on the blue curve which represents the sample of the reactor effluent. Comparison between the two plots confirms the disappearance of these groups. (Figure 19) plots a contrast of FTIR of MV dye compared with tap water after 180 min at optimum operating conditions The Figure at the top illustrates the functional groups in the sample taken at the outlet of the reactor after 180 min., while the Figure at the bottom shows an analysis of drinking water. Obviously the plots confirm that the degradation almost complete.

### Catalyst recuperation

A neutralization process was carried out after photocatalytic treatment for sedimentation of titanium dioxide aggregate. Table 3 presents the turbidity before and after the neutralization process, and Figure 3 was used to estimate the corresponding concentration of TiO_2_. The sediment was washed with H_2_O_2_, dried, and weighted for further use.

**Table 3:** **Variation of turbidity against C**_**TiO2**_ **before and after neutralization process**

***Exp*** **.** ***no*** **.**	${\mathbf{C}}_{{\mathbf{TiO}}_{\mathbf{2}}}$ **(mg/L)**	**Turbidity input (NTU)**	**Residual turbidity (NTU)**
*1*	*200*	*1710*	*285.133*
*2*	*300*	*3300*	*351.845*
*3*	*400*	*5320*	*416.405*
*4*	*500*	*5820*	*459.445*
*5*	*800*	*8690*	*663.885*

### Membrane separation

Low pressure reverse osmosis (LPRO) membrane was used to separate TiO_2_ particles from product water. The experimental results indicated that separation efficiency of LPRO was nearly 100% which suggested the use of such type of membrane for the hyper detoxification process.

Turbidity may be considered as a function of suspended solids concentration in solution. This definition could be used to estimate the separation efficiency of the membrane using equation (10).

10 $$ \mathrm{Seperation}\ \mathrm{efficiency}=\frac{{\left(\mathrm{Turbidity}\right)}_{\mathrm{in}}-{\left(\mathrm{Turbidity}\right)}_{\mathrm{permeate}}}{{\left(\mathrm{Turbidity}\right)}_{\mathrm{in}}} $$

**Figure 20:**
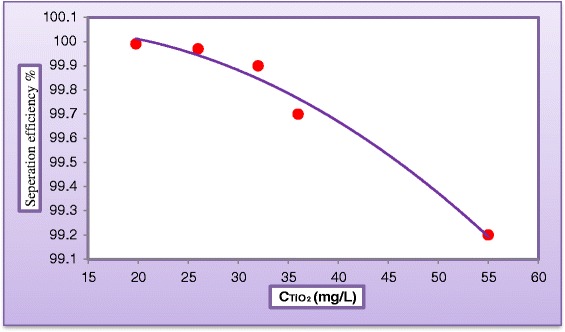
**Variation of membrane separation efficiency against TiO**_**2**_ **concentration in neutralized solution of the reactor effluent.**

**Figure 21:**
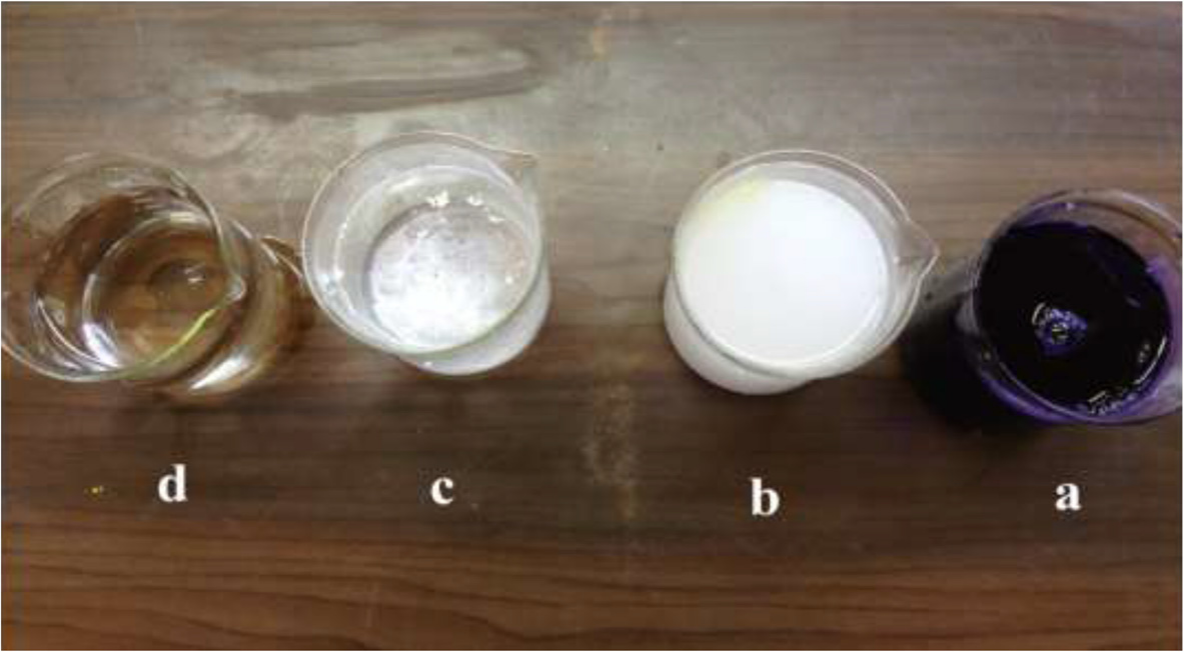
**A sample of wastewater before and after treatment.** A photographic view for a sample of **(a)** synthetic wastewater (dye concentration = 30 mg/L), **(b)** after 180 min treatment by photoreactor, **(c)** after neutralization process, **(d)** after treated by LPRO membrane.

Figure 20 represents the separation efficiency of the LPRO as a function of TiO_2_ particles concentration into a neutralized solution of the photocatalytic reactor effluent. Figure 21 shows a photographic view for a sample of (a) synthetic wastewater (dye concentration = 30 mg/L), (b) after 180 min treatment by photoreactor, (c) after neutralization process, (d) after treated by LPRO membrane.

**Figure 22:**
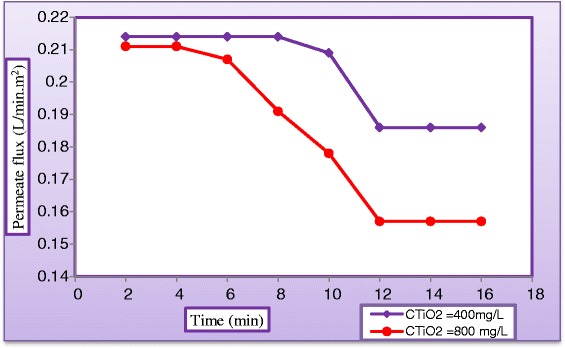
**Variation of permeate flux of LPRO membrane against operating time at different catalyst loadings.**

Despite the strong potential of membrane, one of the common problems encountered in applications is membrane fouling, which can significantly increase the energy consumption of the process over long- term operations [38]. Figure 22 plots the variation in permeate flux (L/min.m^2^) with operating time for solutions of (400 mgTiO_2_/L) and (800 mgTiO_2_/L), respectively. The reduction in permeate flow rate was attributed to the deposition of suspended TiO_2_ particles on the external surface of membrane at its pore openings or within its pores. This deposition became an additional barrier for water to flow through the permeate side of the membrane. Flow decline which could be originated by fouling, was a usual consequence of concentration polarization. Hence, if the feed pressure was held constant, the permeate flow will decrease. Thus, to overcome the additional barrier and to maintain constant permeate flow, the operating pressure must be increased.

## Conclusions

This work focused on the degradation of toxic organic compounds such as methyl violet dye (MV) in water, using a combined photocatalysis/low pressure reverse osmosis (LPRO) system. The performance of the hybrid system was investigated in terms of the degradation efficiency of MV, COD and membrane separation of TiO_2_. The following conclusions have been noticed:

- *Photocatalysis Degradation of Dye*
  - It appears that the flow rate recirculation, irradiation time catalyst load, pH, H_2_O_2_ concentration, and concentration of dye mainly controls the rate of degradation for which optimum conditions for achieving maximum efficiency were established.
  - Regarding catalyst loading, the degradation increased with the mass of catalyst up to an optimum amount. After then the degradation decreased as the catalyst loading continued to increase attributed to the UV light penetration depth which is considerably smaller than in suspension containing optimum amount of catalysts.
  - The capacity of TiO_2_ towards dye degradation strongly depended on pH of suspension. At basic pH, degradation was smaller than that at acidic pH where degradation of anionic dye was fast indicating that the mechanism involving complete mineralization could be achievable.
  - The impact of the liquid Reynolds number on dye degradation shows a positively increasing trend to a point where all the surface of the photocataytic reactor was covered with a thin falling–film of synthetic wastewater, after then the dye degradation started to decrease with further increasing of liquid flow rate. This may be attributed to decreasing the residence time of the reactants.
  - The addition of H_2_O_2_ to TiO_2_ suspension resulted in an increase in the degradation ratio. The H_2_O_2_ acted as electron acceptors to make electron/hole recombination avoided and increased the concentration of ^•^OH radicals.
  - It was found that the kinetics of dye degradation was first order with respect to dye concentration and could be well described by Langmuir-Hinshelwood model with the following rate law. The correlation coefficient (CF) was equal to 0.9892,
    - $$ r=0.0165C/\left(1+0.0209C\right) $$
- *Membrane separation system*
  To make the product water available for reuse, the TiO_2_ particles in suspension must be removed. LPRO membrane was selected to perform the task. The following conclusions can be drawn from the experimental runs.
  - The neutralization process of the reactor effluents seems to be feasible for reduction catalyst loading in the influent line of the membrane system.
  - Increasing the catalyst loading from (400 to 800 mg/L) results in reduction of permeate flow rate by 15%.
  - The TiO_2_ separation efficiency of membrane could be estimated by utilizing the turbidity values of influent and effluent solutions.
  - The LPRO membrane system has proved to be an efficient solution for the separation of TiO_2_ suspended particles.
